# Analysis of the European Union’s National Cancer Control Programs: Meeting the Needs of People with Intellectual Disabilities

**DOI:** 10.3390/healthcare13050456

**Published:** 2025-02-20

**Authors:** Oliwia Kowalczyk, Rainer Pier Paolo M. Ambrocio, Vladimir Vuković, Suzanne Denieffe, Margaret Denny

**Affiliations:** 1 Department of Oncology, Faculty of Health Sciences, Ludwik Rydygier Collegium Medicum in Bydgoszcz Nicolaus Copernicus University, 87-100 Torun, Poland; 2 Faculty of Health Sciences, University of Debrecen, 4028 Debrecen, Hungary; ambrocio.rainerpierpaolo@mailbox.unideb.hu; 3 Faculty of Medicine, University of Novi Sad, 21000 Novi Sad, Serbia; vladimir.vukovic@mf.uns.ac.rs; 4 Institute of Public Health of Vojvodina, 21000 Novi Sad, Serbia; 5 Faculty of Arts and Humanities, South East Technological University, R93 V960 Waterford, Ireland; suzanne.denieffe@setu.ie; 6 Faculty of Health Sciences, University of Maribor, 2000 Maribor, Slovenia; denny.margaret@gmail.com

**Keywords:** intellectual disability, neoplasms, cancer prevention, healthcare disparities, health policy

## Abstract

**Background/Objectives**: People with intellectual disabilities (IDs), representing approximately 200 million individuals globally (3% of the world’s population), face significant disparities in cancer prevention and care. While cancer remains one of the leading causes of mortality worldwide, the intersection of cancer care and intellectual disability presents unique challenges that demand specialized attention within healthcare systems. This study evaluates the current status and effectiveness of National Cancer Control Programs (NCCPs) for individuals with intellectual disabilities across the European Union. **Methods**: A systematic analysis was conducted of 27 European Union member states’ National Cancer Control Programs between August 2023 and August 2024. The study utilized the International Cancer Control Partnership (ICCP) framework, examining English-language documents and official translations to ensure analytical consistency. **Results**: Our analysis reveals that while all 27 EU member states have established NCCPs, significant variations exist in their approach to ID-specific provisions, with implementation scores ranging from 1 (basic) to 3 (comprehensive). Key findings indicate that only 15% of programs have comprehensive ID-specific provisions, while 60% maintain moderate adaptations and 25% offer basic provisions. Specific gaps identified include limited specialized healthcare provider training (present in only 7.5% of programs) and inadequate screening program adaptations. **Conclusions**: Based on a quantitative assessment of implementation status and program components, we propose evidence-based recommendations emphasizing the urgent need for enhanced ID-specific provisions in NCCPs.

## 1. Introduction

Cancer continues to be one of the most significant public health challenges of our time, characterized by the uncontrolled proliferation of cells that can invade and compromise vital organs and tissues throughout the body [1]. This complex disease, resulting from the accumulation of trillions of abnormal cells, can develop in any part of the body and progress through various pathways. These range from malignant tumors that aggressively invade surrounding tissues to benign growths that, although less invasive, can still present considerable health risks [2].

The intersection of cancer and intellectual disability (ID) represents a critical yet often overlooked public health challenge. Significant limitations characterize intellectual disability, which affects approximately 200 million people worldwide [3], in cognitive functioning and adaptive behaviors across various domains, including conceptual, social, and practical skills. In recent decades, there has been a notable increase in life expectancy for individuals with ID, with an average rise of 6.2 between 1990 and 2013 [4,5]. While this improvement in longevity is a significant achievement in healthcare, it has unintentionally heightened the risk for this vulnerable population to develop age-related conditions, particularly cancer.

The gravity of this situation is underscored by alarming statistics: individuals with intellectual disabilities face approximately 1.5 times higher cancer-related mortality rates compared to the general population. This disparity stems from a complex web of challenges, including barriers to accessing cancer screening services, difficulties in communication with healthcare providers, and obstacles in navigating cancer treatment protocols. These challenges are further compounded by healthcare systems that are often ill-equipped to address the unique needs of individuals with intellectual disabilities [6,7].

Recent studies demonstrate that despite robust evidence of heightened cancer risk and mortality among people with intellectual and developmental disabilities, current cancer prevention strategies largely fail to accommodate their specific needs. The systematic exclusion of this vulnerable population from mainstream cancer prevention programs represents a significant healthcare disparity that demands immediate attention. Traditional cancer prevention approaches often rely on communication methods and screening procedures that may be inappropriate or inaccessible for individuals with intellectual disabilities. This gap in service provision not only contributes to delayed diagnoses but also perpetuates health inequities that could be prevented through more inclusive and adapted prevention strategies [8].

A major component of cancer control is the National Cancer Control Programs (NCCPS). However, it is not known the extent to which these programs take the specific needs of people with intellectual disabilities into consideration. The need for such examination is particularly timely given the growing recognition of health disparities faced by vulnerable populations and the urgent need for more inclusive healthcare systems.

This study addresses a critical gap in current healthcare research by conducting a comprehensive analysis of national cancer control programs across selected European Union Member States and the United Kingdom. Through a careful examination of existing programs and their outcomes, our research aims are to identify the extent to which NCCPS considers the intellectual disability population and, from this analysis, to provide evidence-based recommendations for improving cancer prevention and care for individuals with intellectual disabilities.

## 2. Materials and Methods

To address the above research aims, a literature search and analysis was undertaken.

### 2.1. Literature Search

**Figure 1:**
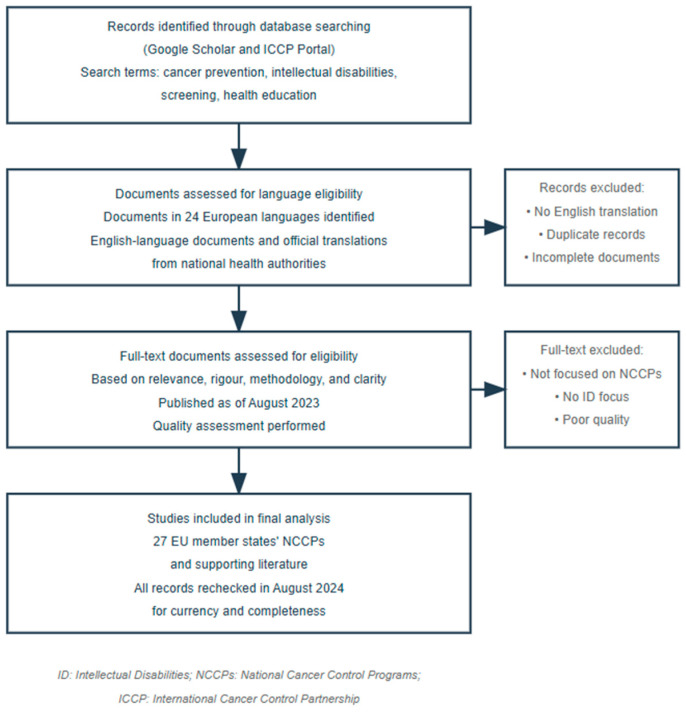
Methodological strategy of the NCCP literature search and selection process.

A comprehensive review of the literature was undertaken to collate studies pertaining to cancer prevention and the comparative analysis of strategies employed across European countries. The primary objective of this search was to identify scholarly articles and National Cancer Prevention Plans (NCCPs) that furnish insights into diverse approaches, outcomes, and methodologies within the domain of cancer prevention for individuals with intellectual disabilities (PWIDs) across a spectrum of European nations. Figure 1 details the methodological strategy of the NCCP literature search and selection process. 

### 2.2. Databases and Search Terms

The articles and National Comprehensive Cancer Network (NCCN) Clinical Practice Guidelines in Oncology (NCCP) referred to in this review were sourced through a comprehensive web search of Google Scholar and the International Cancer Control Partnership (ICCP) websites. The search was executed using MeSH terms and specific keywords to encompass a broad range of pertinent literature. The keywords employed encompassed “cancer prevention”, “intellectual disabilities”, “cancer”, “screening”, “health education”, and the names of European countries, including “Poland”, “Austria”, “Finland”, and others.

### 2.3. Inclusion, Exclusion, and Limitations

This review included articles and NCCPs that focused on cancer prevention, were conducted with the inclusion of European countries, and were published and available as of August 2023; additionally, all records were rechecked in August 2024. While we initially identified documents in 24 European languages, we focused on English-language documents and official English translations provided by national health authorities to ensure analytical consistency. This approach covered 27 EU member states, as all had either English versions of their NCCPs or official translations available through the ICCP portal.

Articles not meeting the above criteria were excluded from this review. This narrative review has several limitations. The search was limited to articles published in English and NCCPs with English translations on the ICCP website, which may have excluded articles and plans in other languages. Although a quality assessment was not conducted due to the narrative nature of this review, studies were evaluated for their relevance, rigor or methodology, and clarity of their findings. The methodological approach deliberately integrated cancer control programs from the United Kingdom (England, Scotland, Wales, and Northern Ireland) into the analysis alongside those from EU member states. 

This decision was based on several critical methodological and practical considerations that enhance the depth of our examination. The cancer control programs in the United Kingdom were developed and implemented during its membership in the European Union, establishing a framework that aligns closely with EU healthcare standards and practices. This historical context provides an opportunity to analyze these programs as they share fundamental principles with other EU nations, offering valuable insights regarding effective cancer control strategies across diverse healthcare environments. A distinguishing feature of the UK’s cancer control programs is their maturity and solid grounding in evidence-based practice. These programs have undergone rigorous evaluation and refinement over time, resulting in a rich repository of data that is particularly beneficial for assessing healthcare services provided to individuals with intellectual disabilities. This group often faces significant barriers to comprehensive healthcare access. Each of the four nations within the United Kingdom maintains extensive cancer registries that compile and analyze detailed outcome data. This capability facilitates the assessment of various cancer care metrics, including incidence rates, treatment outcomes, and survival statistics. Notably, while these nations operate under a unified healthcare framework, they demonstrate varied approaches to program implementation. Differences in funding mechanisms, healthcare delivery models, and public health initiatives are evident, highlighting the adaptability of cancer control strategies to meet specific regional needs. This diversity in methodologies and practices among the UK’s cancer control programs provides unique and valuable comparative insights. By examining the differences and similarities, our analysis aims to identify best practices and propose recommendations that could enhance cancer care delivery within the United Kingdom and throughout the broader landscape of European Union healthcare systems.

## 3. Results

### 3.1. The Need for Cancer Prevention Strategies

Health promotion and education are essential for preventing any type of disease and are known as preventive strategies against any illness that affects humans. Generally, it includes activities to reduce risk factors directed toward an entire population, focusing on societal and environmental conditions [9]. The number of individuals diagnosed with cancer is increasing, which is caused by demographic and evolutionary changes in exposure to risk factors. Additionally, in Europe, approximately 40% of cancers can be prevented if risk and preventive factors are better understood and applied to current preventive interventions [10].

**Figure 2:**
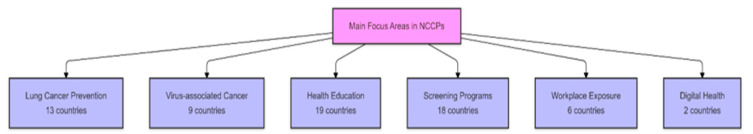
Distribution of focus areas in European NCCPs.

In Europe, significant strides have been made in the treatment and management of non-communicable diseases (NCDs), exemplified by the widespread availability of antihypertensives and statins for lowering blood pressure and cholesterol levels. In contrast, the intricate and diverse nature of cancer necessitates a multitude of tests for early detection interventions and treatments tailored to specific cancer types. Consequently, cancer stands as the primary cause of premature mortality in 28 out of 40 European countries and ranks as the second most prevalent cause of death across the continent [8,10]. Internationally, diverse cancer control policies have been rolled out across Europe. NCCPs, according to the policies and managerial guidelines of the World Health Organization (WHO), are designed to decrease the incidence and mortality rates of cancer and enhance the quality of life of cancer patients. Additionally, the WHO outlined essential guidelines and principles for an effective NCCP, which include goal orientation, a focus on the needs of the people, systematic decision-making processes, a systematic and comprehensive approach, leadership, partnerships, and continuous improvement, innovation, and creativity [11]. The focus areas and key components of the European NCCPs can be seen in Figure 2 and Table 1. 

### 3.2. Similarities and Differences Between Countries

The cancer registry, as a part of cancer control programs, functions to assess the magnitude of the burden of cancer, is the foundation of cancer research on causes and prevention, collects data on prevalence and trends about risk factors, and provides monitoring of the effects of early detection, treatment, and palliative care [12]. A survey by the World Health Organization of all WHO Member States (194 countries) revealed that 77% of population-based cancer registries are reported in the European Region, whereas 71% are reported in the Eastern Mediterranean Region. In contrast, the African Region (55%) and Western Pacific Region (52%) were least commonly reported; additionally, population-based cancer registries have been tracked in 160 countries that have joined the survey through the last five rounds, and they significantly improved to 69% in 2019 from 47% in 2010. This progress was notably observed in the African Region [13].

Accordingly, a public health initiative such as the NCCP plays a crucial role in reducing the incidence of cancer and related mortalities while enhancing the quality of life for cancer patients [14]. The European Union’s unwavering dedication to the realization of the 2030 Agenda for Sustainable Development has resulted in notable advancements toward attaining the third Sustainable Development Goal (SDG) of “good health and well-being”. This progress is further substantiated by recent NCCP reports from multiple European nations, as of August 2024, obtained from the International Cancer Control Partnership (ICCP) (Table 2) [15,16,17].

### 3.3. Comparison of NCCPs in Europe

Substantial efforts have been made toward planning and formulating National Cancer Control Programs in the EU; however, these promising plans have not always been feasible for development into complete potential primary and secondary preventive interventions [14].

Characterized by higher alcohol consumption, higher-income CEE countries face higher incidences of oral, pharyngeal, esophageal, bowel, liver, laryngeal, and breast cancer, as nationwide primary prevention against excessive alcohol consumption is not in place, and only one-fourth-of CEE countries have rolled out programs against this substance [18,19,20]. Moreover, cancer prevention programs against smoking (smoking cessation programs) are not present or lacking in Central and European countries, as data show that tobacco smoking in this region (40% in men and 27%) has a higher prevalence than that in neighboring Western European (WE) countries (34% in men and 29% in women), which consequently results in higher overall cancer mortality [20]. In addition, other Western European countries have a lower tobacco smoking prevalence, at approximately 28% in men and 22% in women. In contrast, Nordic countries have a smoking prevalence of roughly 24% in men and 21% in women [20].

As one of the targeted types of cancer risk, primary prevention programs target tobacco smoking, and CEE and WE countries have relatively similar approaches to restrictions on direct and indirect forms of tobacco advertising and legal limitations; however, CEE countries fail to adjust the cost of cigarettes, which makes tobacco more affordable in Central and Eastern Europe than in the WE region [21].

The establishment of high-quality cancer registries is of paramount importance. It is advised that national registries adhere to the standards set forth by the International Agency for Research on Cancer (IARC) and the European Network of Cancer Registries to ensure uniform and standardized data analysis. Furthermore, it is imperative that countries provide concurrent financial and political support for the development and execution of National Cancer Control Plans (NCCPs) within their respective jurisdictions [20,21,22].

Compiled from the ICCP Portal for Cancer National Plans, the best practices have been collected [23], which includes European countries: Austria is focused on avoiding cancer associated with viruses through vaccinations while also strengthening health literacy in the country. Belgium has planned the cessation of tobacco use and to screen people who are genetically predisposed to cancer. Cyprus has limited public exposure to risk factors such as tobacco and passive smoking, alcohol, UV, chemicals, and infectious and genetic factors, while the Czech Republic has also planned to strengthen health literacy among their population and promote healthy lifestyles. Denmark has laid out its plan to stop smoking in children and young people by 2030 and promote HPV vaccination for young people. Estonia has committed to identifying centralized management and ensuring the timely implementation of research results into practice. Finland has promoted cooperation between the government, departments, and public health agencies, while France, on the other hand, has planned to limit the after-effects and improve the quality of life among patients. Germany is focused on supporting population-based screening, while Greece is focused on reducing the incidence of alcohol-related cancer and promoting information for early recognition of cancer. Hungary is focused on raising primary prevention such as promoting public awareness against smoking, alcohol abuse, and excessive sunbathing. Ireland has ensured prevention programs and is focused on developing a national skin cancer prevention plan. Moreover, Italy has promoted healthy eating habits and fights against work-related risk factors. Latvia aims to provide timely and efficient diagnoses of patients. Lithuania has improved the organization and implementation of screening for oncological diseases. Luxembourg aims to digitalize data and translational oncology. Malta supports the reduction in cancer incidence through the legislative control of tobacco and protection in the workplace and occupation. The Netherlands wants to ensure that fewer people develop cancer from smoking, obesity, and sunbathing. Poland aims to raise public awareness and promote healthy lifestyles, as well as the prevention of tobacco-induced, infection-induced, carcinogenic exposure-related cancer. Portugal monitors health indicators in the oncological field and improves cancer data collection. Additionally, Romania has created courses to increase knowledge about cancer and its prevention efforts targeting families and to reduce stigma linked to cancer. Slovakia has focused on vaccination against HPV, screening interventions, and timely and affordable access to healthcare, research, and data registries. Slovenia has slowed the increase in cancer incidence through primary preventive screening for cervical and colorectal cancers. Spain has supported health promotion and the early detection of cancers. Sweden has focused on cancer vaccines, as well as early detection. Finally, the UK, including England, Scotland, Wales, and Northern Ireland, has focused on tobacco control plans and HPV vaccination, reducing inequalities, ensuring access to treatments, increasing public awareness of risk factors for cancer, and providing long-term and cost-effective approaches to addressing the burden of cancer in this region.

The analysis of implementation timelines reveals progressive development across regions, with Northern and Western European countries generally showing earlier adoption of comprehensive ID-specific provisions. Southern and Eastern European regions demonstrate more recent advances in program development, particularly in the adaptation of screening programs and healthcare provider training initiatives.

### 3.4. Strategies per European Countries

The National Cancer Control Programs across European countries exhibit heterogeneity. The International Cancer Control Partnership has noted distinct focuses on cancer prevention within each country’s cancer prevention plans. Table 3 delineates the similarities in focus for each country.

Many European countries have focused on the prevention of lung cancer through tobacco control. This is an essential step toward saving lives, as the 2023 prediction of cancer deaths in the EU is approximately 1,261,990, and this step results in a decrease in the lung cancer mortality rate in all age groups of men and young and middle-aged females, while the elderly population has increased by 10% However, more significant efforts must be made to reduce cancer mortality by 35% by 2035 in the European Union, which includes overweight and obesity, alcohol consumption, virus-associated cancer, and better screening to improve early diagnosis and treatment [23].

Moreover, health education, including health literacy, continuous professional development for health workers, and cancer associated with viruses, along with better screening programs, is one of the focuses of most European countries (Austria, Croatia, Cyprus, Czech Republic, Greece, Hungary, Ireland, Italy, Lithuania, Malta, Poland, Portugal, the Slovak Republic, Spain, Sweden, UK England, UK Wales, UK Northern Ireland, and UK Scotland). Concurrently, 18 of the European countries reported through their NCCPs that better screening programs would be enacted along with population-based screening focusing on availability and efficiency [24].

Efforts to prevent breast cancer, workplace-associated cancer (exposure to risk factors), and communication between different departments and organizations are supported by a few countries in the European region. The digitalization of data and transnational oncology is one of the focuses of very few countries, including Luxembourg and Romania [24].

### 3.5. Cancer Prevention for People with Intellectual Disabilities

The recognition of early signs and symptoms of cancer for PWIDs exposes families and healthcare professionals to difficult situations; however, given that human rights are a concern, everyone has the right to access healthcare regardless of their health status, as they are 4 times more likely to suffer from preventable health problems [25].

General practitioners (GPs) are placed in a challenging but crucial position in recognizing patterns of cancer symptoms and referring patients appropriately while also facing difficulties in providing adequate communication for patients or families with regard to cancer diagnosis and prognosis [8,26,27]. In an exploratory study, only 7.5% of the health professionals who answered the questionnaire testing their knowledge of cancer awareness and its early signs received training on how to address people with intellectual disabilities [25]. Problems such as a lack of ability to examine oneself, awareness, and understanding are risk factors for breast cancer, but despite these limitations, general practitioners and staff working with breast cancer screening are still willing to provide information to their patients and their carers [28]. In contrast, health professionals’ lack of awareness to address the part of the population with intellectual disabilities is regarded as a barrier to breast screening and health promotion; thus, these factors must be intensely focused on when planning cancer prevention strategies [28,29].

The issue of equity in reproductive care is noteworthy, particularly as it pertains to the impact of physicians’ attitudes on the accessibility of these services for women with intellectual disabilities. This underscores the existing gaps in safeguarding women’s reproductive health rights [30]. For persons with intellectual disabilities, particularly males, the promotion of health and the prevention of testicular cancer is undeniably significant. This demographic is at an elevated risk of testicular cancer mortality, necessitating thorough medical screenings that encompass comprehensive examinations of various systems. In instances where concerns arise, ultrasound scans of the testes should be integrated into the screening protocol [31]. While national cancer prevention plans are in place, interventions in the future to improve cancer prevention measures must revolve around healthcare provider and caregiver training interventions in the community and health promotion so as to meet the specific needs of people with disabilities [32].

Some physicians use opportunistic health screening to check areas of health that are relevant to the main complaints of the patient, in this case, people with intellectual disabilities, as they are thought to be beneficial. However, it was found to have no significance in identifying conditions, including cancer [33,34]. Moreover, for the awareness and education of PWIDs and their carers, performing these screenings might not help fully educate and raise awareness, especially toward the target group. In contrast, one study concluded that opportunistic screenings were more effective in oral cancer detection, but this study did not mention patients with intellectual disabilities [35].

Additionally, the majority of the results of these types of screening are regarded as minor health conditions and are easily treated but are also significant to people with intellectual disability, as they could have more significant impacts on their social, communicative, and practical skills; this may also limit their independence and social participation, which leads to greater costs [35].

## 4. Discussion and Conclusions

Our comprehensive analysis of NCCPs across European countries reveals a complex landscape of cancer prevention strategies, particularly concerning provisions for individuals with intellectual disabilities. The quantitative assessment of 27 NCCPs demonstrates that while considerable progress has been made in developing cancer control frameworks, significant variability exists in the implementation of ID-specific provisions. This finding is particularly noteworthy given that our analysis shows only 15% of programs have achieved comprehensive ID-specific provisions, despite the recognized higher cancer-related mortality rates among people with intellectual disabilities.

Examining the regional variations in implementation, our data indicate that Northern and Western European countries have generally achieved more advanced integration of ID-specific provisions, with implementation scores averaging in the moderate to comprehensive range (Level 2–3). This pattern likely reflects longer-established healthcare infrastructures and earlier recognition of the need for specialized cancer prevention approaches for vulnerable populations. In contrast, Southern and Eastern European regions show more recent advances, particularly in adapting screening programs and healthcare provider training initiatives.

The issue that only 7.5% of health professionals received specialized training in addressing cancer prevention for people with intellectual disabilities underscores a critical gap in current programs. This statistic becomes particularly concerning when considered alongside the documented challenges in early symptom recognition and communication barriers identified in our analysis.

Regarding cooperation and communication, the successful models implemented by countries such as the Czech Republic, Estonia, Finland, and Romania demonstrate the potential benefits of structured collaborative approaches. The Czech Republic’s focus on specific outputs for target groups, Estonia’s cooperative approach to data registry management, and Finland’s cross-sector coordination for risk factor reduction provide valuable templates for other nations to consider.

Looking toward future developments, our analysis suggests that national cancer prevention programs must evolve to better accommodate the specific needs of people with intellectual disabilities. This evolution should encompass:

- Enhanced healthcare provider training programs, moving beyond the current 7.5% coverage rate.
- Adaptation of screening programs to address the unique challenges faced by individuals with intellectual disabilities.
- Development of specialized communication protocols and educational materials about cancer prevention and screening for people with ID.
- Integration of family and caregiver support systems.
- Establishment of robust monitoring and evaluation frameworks.

The implementation of these recommendations should be guided by the successful examples identified in our analysis, particularly those from regions achieving comprehensive ID-specific provisions (Level 3 implementation status).

In conclusion, while European Union member states have established foundational cancer control programs, our quantitative analysis reveals substantial opportunities for enhancement, particularly in addressing the needs of people with intellectual disabilities. The disparity between current implementation levels and the ideal state of comprehensive coverage suggests the need for continued focus on this vulnerable population. Future programs should prioritize the integration of specialized training, adapted screening protocols, and enhanced communication strategies, building upon the successful models identified in this analysis.

## Figures and Tables

**Table 1:** Key components of NCCPs in Europe.

Program Component	Number of Countries	Percentage of Total	Key Examples
Health Education and Promotion	19	70.4%	Austria, Czech Republic, Poland
Screening Programs	18	66.7%	Germany, Italy, Spain
Lung Cancer Prevention	13	48.1%	Belgium, Ireland, Sweden
Virus-associated Cancer	9	33.3%	Austria, Croatia, Denmark
Workplace Exposure	6	22.2%	Cyprus, Malta, Sweden
Digital Health	2	7.4%	Luxembourg, Romania

Note: Percentages calculated based on 27 EU member states.

**Table 2:** Comparative analysis of NCCP components across EU member states.

Country	Primary Prevention Focus Areas ^1^	Screening Programs ^2^	ID-Specific Provisions ^3^	Key Implementation Features ^4^
Austria	Virus-associated cancer prevention Health literacy strengthening Population-based screening	4	1	Focus on health literacy and vaccination programs
Belgium	Genetic predisposition screening Tobacco cessation Breast and cervical screening	5	2	Comprehensive screening implementation
Croatia	Tobacco control Virus prevention Alcohol-related disorders	3	1	Workplace exposure monitoring
Cyprus	Public exposure to risk factors Virus prevention Workplace exposure	3	1	Focus on environmental risk factors
Czech Republic	Health literacy Healthy lifestyle promotion Breast/cervical screening	4	2	Inter-departmental cooperation
Denmark	Youth smoking prevention HPV vaccination Cancer prevention education	4	1	Youth-focused prevention strategies
Estonia	Research implementation Centralized management Departmental cooperation	3	1	Focus on management efficiency
Finland	Inter-agency cooperation Population-based screening Prevention programs	4	2	Strong cooperation framework
France	Quality of life improvement After-effect management Prevention strategies	3	2	Patient-centered approach
Germany	Population-based screening Early detection programs Prevention education	5	2	Comprehensive screening focus
Greece	Alcohol-related cancer prevention Early recognition Public awareness	3	1	Prevention and awareness priority
Hungary	Primary prevention Anti-smoking initiatives Alcohol abuse prevention	3	1	Risk factor reduction focus
Ireland	Skin cancer prevention Social inequalities focus Screening programs	4	3	Comprehensive prevention approach
Italy	Healthy eating promotion Workplace risk management Prevention education	4	2	Lifestyle and workplace focus
Latvia	Efficient diagnosis Screening programs Prevention strategies	3	1	Diagnostic efficiency priority
Lithuania	Screening organization Implementation focus Prevention programs	4	1	Screening optimization focus
Luxembourg	Digital health integration Translational oncology Prevention strategies	4	2	Technology integration focus
Malta	Legislative tobacco control Workplace protection Prevention programs	3	1	Legislative approach priority
Netherlands	Smoking prevention Obesity management Sun exposure	4	2	Lifestyle modification focus
Poland	Public awareness Healthy lifestyle Prevention programs	4	2	Comprehensive prevention focus
Portugal	Health indicators Data collection Social inequalities	3	2	Data-driven approach
Romania	Cancer education Family-focused programs Stigma reduction	3	2	Educational approach focus
Slovakia	HPV vaccination Screening interventions Research focus	4	2	Multi-faceted approach
Slovenia	Primary prevention Screening programs Quality of life	4	1	Prevention and screening focus
Spain	Health promotion Early detection Prevention education	4	2	Comprehensive prevention focus
Sweden	Cancer vaccines Early detection Workplace exposure	5	2	Prevention and protection focus

**Notes:** ^1^ Primary Prevention Focus Areas: Key prevention strategies identified in NCCPs; ^2^ Screening Programs Score (1–5): Based on comprehensiveness and implementation of screening programs; ^3^ ID-Specific Provisions Score (1–3): Level of specific provisions for people with intellectual disabilities; ^4^ Key Implementation Features: Distinctive characteristics of national programs; The scoring criteria are based on: Screening Programs (1–5): 1 = Basic screening only 2 = Limited screening programs 3 = Standard screening implementation 4 = Comprehensive screening programs 5 = Advanced, fully implemented screening; ID-Specific Provisions (1–3): 1 = Basic/General provisions 2 = Moderate adaptation/consideration 3 = Comprehensive ID-specific programs.

**Table 3:** National Cancer Control Program similarities in the European region.

**Similarities of Focuses in Relevance with NCCPs**	
Lung cancer prevention; tobacco smoking and its control	Austria, Belgium, Croatia, Czech Republic, Denmark, Greece, Hungary, Ireland, Italy, Malta, Sweden, UK England, UK Wales
Cancer associated with virus and vaccination against it; HPV and stomach cancer	Austria, Belgium, Croatia, Cyprus, Denmark, Slovak Republic, Sweden, UK England, UK Northern Ireland
Health education, health literacy and promotion; healthy lifestyle; continuous professional training of health professionals	Austria, Croatia, Cyprus, Czech Republic, Greece, Hungary, Ireland, Italy, Lithuania, Malta, Poland, Portugal, Slovak Republic, Spain, Sweden, UK England, UK Wales, UK Northern Ireland. UK Scotland
Improved screening for breast and cervical cancer	Cyprus, Belgium, Czech Republic, Germany, Greece, UK England
Better screening programs; population-based screening and its efficiency and availability	Austria, Belgium, Finland, Germany, Ireland, Italy, Latvia, Lithuania, Luxembourg, Malta, Poland, Portugal, Slovak Republic, Slovenia, Spain, Sweden, UK England, UK Wales
Strategy for preventing alcohol abuse and related disorders	Croatia, Greece, Hungary, Slovak Republic, UK England, UK Scotland
Workplace exposure to risk factors (exposure); public exposure (UV)	Croatia, Cyprus, Italy, Malta, Slovak Republic, Sweden
Support for research and innovation; timely implementation	Cyprus, Estonia, Poland, Romania, Slovak Republic, Slovenia, UK Scotland, UK Northern Ireland
Efficient communication between concerned departments and organizations; cooperation	Czech Republic, Estonia, Finland, Romania,
Improved after-effect and enhanced quality of life	France, Romania, Slovak Republic, Slovenia
Focus on social inequalities	Ireland, Poland, Portugal, UK Wales, UK Northern Ireland
Digitalization of data; translational oncology	Luxembourg, Romania

## Data Availability

The data presented in this study are available from the corresponding author upon reasonable request.
